# Lever reduction using polyaxial screw and rod fixation system for the treatment of degenerative lumbar spondylolisthesis with spinal stenosis: technique and clinical outcome

**DOI:** 10.1186/s13018-015-0168-x

**Published:** 2015-02-15

**Authors:** Zu-De Liu, Xin-Feng Li, Lie Qian, Lian-Ming Wu, Li-Feng Lao, Han-Tao Wang

**Affiliations:** Department of Orthopaedic Surgery, Ren Ji Hospital, Shanghai Jiao Tong University School of Medicine, Shanghai, China; Department of Radiology, Ren Ji Hospital, Shanghai Jiao Tong University School of Medicine, Shanghai, China

**Keywords:** Degenerative spondylolisthesis, Lumbar spine, Reduction, Technique

## Abstract

**Background:**

The management for degenerative lumbar spondylolisthesis with spinal stenosis remains controversial. Reduction of lumbar spondylolisthesis has been performed via numerous techniques. Most of them need extra reduction assembly.

**Methods:**

In this retrospective analysis, 27 patients of degenerative lumbar spondylolisthesis with spinal stenosis underwent reduction using polyaxial screw and rod constructs and posterolateral fusion. The average age at the time of surgery was 53 ± 3.23 years. The outcome measures consisted of a radiographic assessment of deformity and fusion rate and a clinical assessment of perioperative improvement in low back pain and function. Preoperative and postoperative radiographic evaluation included the percent slip, slip angle, and the lumbar lordosis between L1 and the sacrum measured using the Cobb method. Before surgery and at the final follow-up, the Oswestry Disability Index (ODI) and the visual pain analog scale (VPAS) between 0 (no pain) and 10 (maximal pain) were quantified.

**Results:**

The average follow-up period more than 5 years was available. The mean operative time was 90.19 ± 14.51 min, and the mean blood loss during surgery was 152.59 ± 45.71 ml. The mean length of incision was 4.83 ± 0.63 cm. The average percent slippage and the mean slip angle were, respectively, 19.8 ± 4.49% and 9.69 ± 3.79° before surgery, 5.09 ± 3.40% and 6.39 ± 3.16° after surgery, and 5.67 ± 3.92% and 7.21 ± 3.05° at the last follow-up. The average lumbar lordosis was 36.88 ± 2.64° before surgery, 41.96 ± 1.64° after surgery, and 40.27 ± 1.19° at the final follow-up. No neurologic deficit occurred. Solid fusion was achieved for all cases. Compared with the outcome preoperation, the data improved from 6.56 ± 1.40 to 2.48 ± 1.16 for VPAS pain scores and from 32.22 ± 3.57 to 10.93 ± 4.93 for the ODI at the final follow-up.

**Conclusions:**

Lever slip reduction maneuver techniques using polyaxial screw and rod fixation system was simple and practicable. The treatment outcomes showed satisfactory radiographic characteristics and clinical results. The length of the incision was relatively small with a low intraoperative blood loss and short operation time.

## Introduction

Degenerative lumbar spondylolisthesis with spinal stenosis is a common condition of the aging spine. The management remains controversial. Regarding the long-term effects, recent studies demonstrated that, compared with nonoperative treatment, surgical treatment could achieve greater pain relief and improvement in function when the results were followed over 2 years [1] and 4 years [2]. There are a variety of surgical methods that have been used for the management of degenerative spondylolisthesis, including posterolateral *in situ* fusion, posterolateral instrumented fusion with pedicle screws, fusion with transforaminal lumbar interbody grafts, anterior lumbar interbody fusion, posterolateral instrumented fusion with pedicle screws plus interbody fusion, and dynamic stabilization [3]. The North American Spine Society’s guideline recommended that the optimal surgery is decompression with an instrumented intertransverse process fusion [4,5]. However, the current studies in the literature could not identify the best surgical technique to perform for lumbar degenerative spondylolisthesis.

The development of surgical techniques and instrumentation provide a practical way for spondylolisthetic deformity reduction and spinal balance restoration. The possible benefits of reduction of lumbar degenerative spondylolisthesis have not been adequately studied. Segmental imbalance may be a factor influencing the later development of adjacent segment disease [6]. The sagittal balance could significantly affect low back pain in patients undergoing posterior decompression and instrumented fusion for degenerative lumbar spine disease [7]. Sagittal deformity correction might improve short- and long-term outcomes for patients with degenerative lumbar spondylolisthesis [8].

Reduction of lumbar spondylolisthesis has been performed via a variety of techniques including Harrington rod distraction [9,10], posterior reduction by instrumented segments [11,12], and the recently reported insert-and-rotate spacer technique [8,13]. Polyaxial pedicle screw system is designed to be more versatile. This system is adjustable to connect the rod and secure the head to the pedicle screw. The purpose of this study was to review our lever distraction and reduction experience using polyaxial screw and rod fixation system with respect to radiographic and clinical outcomes.

## Material and methods

Between 2005 and 2011, 27 patients of degenerative lumbar spondylolisthesis with spinal stenosis underwent reduction using polyaxial screw and rod fixation system and posterolateral fusion. The average age was 53 ± 3.23 years. Twenty-two were females, 5 males. No patients had previous lumbar surgeries. The patients with pathologic conditions of the lumbar spine such as trauma, tumor, or infection were excluded from this study. The mean duration of preoperative symptoms was 6.07 ± 4.05 months. Radiological evaluation showed that spondylothesis located at L4–5 in 22 patients and L5–S1 in five patients.

### Deformity assessment and clinical outcome measures

**Figure 1:**
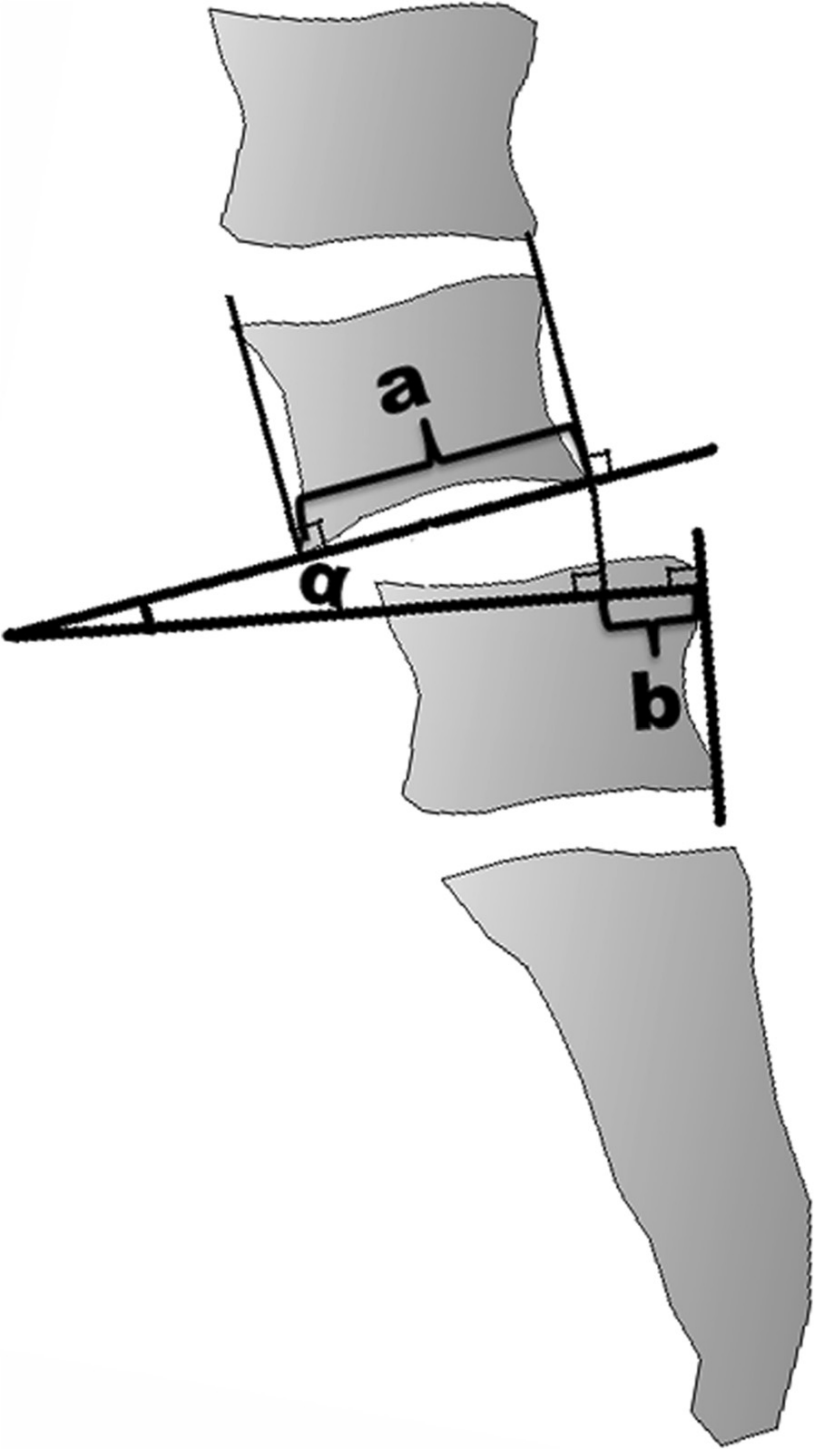
**Methods of the percent slip and the slip angle assessment.** The amount of slip **(b)** was the distance between the posterior line of the vertebral body below the slipped vertebrae and the paralleled line extended through a posterior rim of the slipped vertebrae. The percent slip was the ratio of b and the anteroposterior dimension of the slipped vertebral body **(a)**. The slip-angle (*α*) was formed by the inferior line of the slipped vertebral body and the line perpendicular to the posterior rim of the vertebrae below the slipped vertebrae.

Preoperative and postoperative radiographic evaluation included the percent slip [14], slip angle, and the lumbar lordosis between L1 and the sacrum measured using the Cobb method [15]. The assessment methods of the percent slip and slip angle were shown in Figure 1. The amount of slip was measured as the distance between the posterior line of the vertebral body below the slipped vertebrae and the paralleled line extended through a posterior rim of the slipped vertebrae (b). The percent slip was defined as the ratio of b and the anteroposterior dimension of the slipped vertebral body (a). The slip angle (*α*) was formed by the inferior line of the slipped vertebral body and the line perpendicular to the posterior rim of the vertebrae below the slipped vertebrae. Before surgery and at the final follow-up, ODI [16] and a visual pain analog scale (VPAS) between 0 (no pain) and 10 (maximal pain) were quantified.

### Operative technique

**Figure 2:**
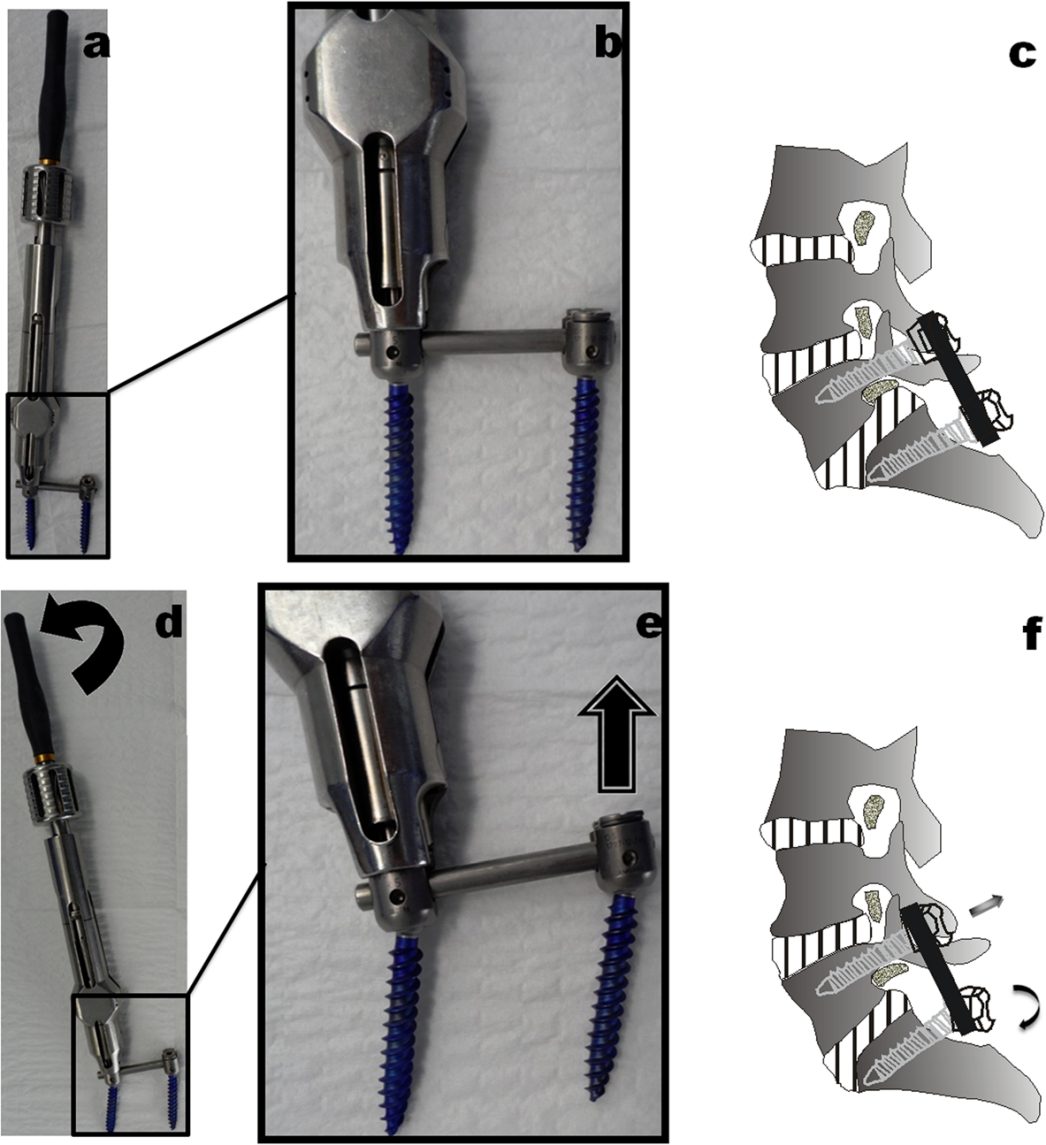
**Polyaxial pedicle screw system (Depuy-AcroMed, Cleveland, OH) was used for reduction and fixation. (a, b, c)** Polyaxial pedicle screws and precontoured rods constructs. Screws were properly tightened but not locked for retaining screw head movement. **(d, e, f)** Lever reduction maneuver technique incorporating segmental distraction and posterior translation forces was performed with bilateral long fixed handle attached to the top-loading polyaxial screws.

After general anesthesia has been administered, the patient was placed prone on a radiolucent operating table. Using standard posterior approach, the level of spondylolisthesis and the neighboring vertebrae intended for stabilization was exposed. Under fluoroscopic control, top-tightening polyaxial pedicle screws were inserted to all levels of the proposed fusion. Then, all patients underwent decompression procedures described by Fitzgerald and Newman [17] at the level of spondylolisthesis. The exuberant masses on the articular joints, the supraspinous and interspinous ligaments, and the ligamentum flavum are removed. The lower half of the laminae of the cephalad vertebrae and medial one third of the inferior articular facet were removed. Bilateral foraminotomy was also conducted for ensuring optimal neurologic safety in the reduction maneuver. Top-tightening polyaxial pedicle screw system was used for reduction and fixation (Figure 2). Appropriately precontoured rods and couplers were assembled over the polyaxial pedicle screws. Then, the screws were properly tightened but not locked for retaining screw head movement (Figure 2a,b,c). Under fluoroscopic control, lever reduction maneuver incorporating segmental distraction and posterior translation forces was performed as shown in Figure 2d,e,f with bilateral long fixed handle attached to the top-loading polyaxial screws. The anatomic alignment was achieved as good as we could. Then, the hex nuts linking the rods to their couplers were locked before the reduction handle device was loosened and removed from the field. Finally, posterolateral bone grafting and fusion were performed, and layered closure was followed. Patients began ambulating on postoperative day 3. Braces were worn for the first 6–8 weeks as they were out of bed. Radiographic characteristics were evaluated at follow-up.

### Statistical analysis

Data were statistically analyzed using a two-tailed paired Student *t* test. Differences were considered statistically significant at *P* ≤ 0.05.

## Results

The average follow-up period was 63.81 months (range from 60 to 70 months). None of the patients were lost to follow-up. The mean operative time was 90.19 ± 14.51 min, and the mean blood loss during surgery was 152.59 ± 45.71 ml. After surgery, no postoperative neurologic complications were recorded. No superficial or deep wound infections were present at the lumbar wound. The operations were completed through small incisions. The mean length of the surgical incision was 4.83 ± 0.63 cm.

### Deformity correction

**Figure 3:**
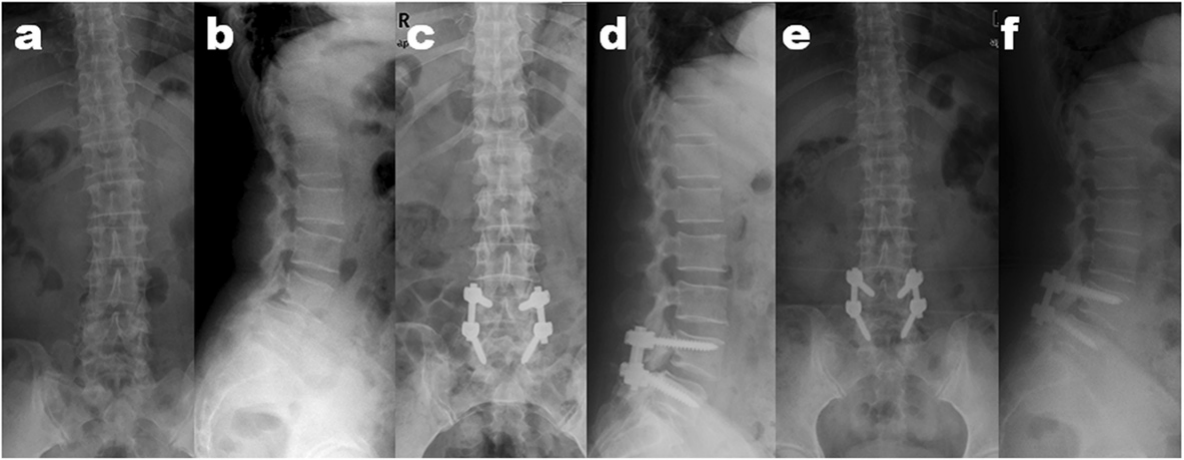
**One patient with a one-level degenerative spondylolisthesis at L4–L5. (a, b)** The preoperative anteroposterior (AP) and lateral radiographs of a 54-year-old woman with lumbar degenerative spondylolisthesis at L4–L5 who had severe claudicant sciatica and back pain. **(c, d)** Immediately after reduction surgery using lever slip reduction maneuver technique with polyaxial screw and rod fixation system through a single posterior surgical approach, AP and lateral views showed that the slip correction was 99.81%. The slip angle and the lumbar lordosis improved from 7.27° and 36° preoperation to 2.74° and 40°, respectively. **(e, f)** At more than 5 years follow-up, the slip correction, the slip angle, and the lumbar lordosis was 99.66%, 4.36° and 38°, respectively.

Deformity correction was assessed using pre- and postoperative X-rays. The average percent slippage was 19.8 ± 4.49% before surgery, 5.09 ± 3.40% after surgery, and 5.67 ± 3.92% at the final follow-up. A significant improvement was indicated for slippage reduction after surgery (*p* < .001) and at the final follow-up (*p* < .001). The slip correction was 71.80 ± 19.38% after surgery and 71.35 ± 12.64% at the final follow-up. The mean slip angle was improved from 9.69 ± 3.79° before surgery to 6.39 ± 3.16° (*p* < .001) after surgery and to 7.21 ± 3.05° at the final follow-up (*p* < .001). The average lumbar lordosis between L1 and the sacrum was 36.88 ± 2.64° before surgery and improved to 41.96 ± 1.64°after surgery (*p* < .001) to 40.27 ± 1.19° at the final follow-up (*p* < .001). At a mean follow-up of 63.81 months, solid union was presented in plain X-rays and no evidence of instrumentation failure was seen. Example of one patient with a one-level degenerative spondylolisthesis at L4–L5 was shown in Figure 3.

### Clinical outcome

Compared with the results before surgery, at the final follow-up, the data improved from 6.56 ± 1.40 to 2.48 ± 1.16 for VPAS pain scores (*p* < .001) and from 32.22 ± 3.57 to 10.93 ± 4.93 for ODI (*p* < .001).

## Discussion

Spondylolisthesis with spinal stenosis often affects older people as degenerative changes progress in the aging population. Slip progression and disc degeneration could lead further as the slip and anterior shear forces increase [18]. The optimal surgical management of lumbar spondylolisthesis remains controversial. Although the reduction of slip remains controversial, the sagittal translation correction in spondylolisthesis is appealing and may be crucial to the prevention of adjacent level degeneration in the long run. Reversal of the lumbosacral deformity could be achieved through reduction maneuvers. In this study, a lever slip reduction maneuver technique using polyaxial screw and rod fixation system through a single posterior surgical approach was introduced. Twenty-seven cases were reviewed. The treatment outcomes showed satisfactory radiographic characteristics and clinical results. No neurologic deficit and pseudarthrosis complication occurred in our series.

In the recent years, the development of surgical techniques and instrumentation provide spinal surgeons with the means to reduce spondylolisthetic deformity and restore spinal balance. Various reduction procedures have been described for lumbar spondylolisthesis. The surgical techniques, including translation reduction with double-threaded screws [19], distract and slip reduction [20], and insert-and-rotate posterior lumbar interbody fusion technique [8,13,21-26] have been reported in the literature. Using a posterior-only approach, the above-described technique could yield substantial deformity correction. More recently, the technical aspects involved in minimally invasive spondylolisthesis reduction have also been described. At L4–5 level, a lateral retroperitoneal transpsoas minimally invasive surgery-lumbar interbody fusion (MIS-LIF) could provide partial reduction of listhesis, and the percutaneous posterior approach was performed with initial locking of the inferior pedicle screw (L-5) and creating a cantilever to allow further reduction [27]. Using translational reduction afforded by the reduction screw extenders, minimally invasive transforaminal lumbar interbody fusion (MI-TLIF) procedure could achieve effective reduction [28,29]. However, extra reduction assembly should be used for most of these reduction techniques. In the present study, reduction could be achieved only through levered forces applied to the screws, rod, and vertebrae using polyaxial pedicle screw and screwdriver with a long handle. Spondylolisthesis and pathologic sagittal rotation correction could be achieved through combined posterior translation and slip manipulation in the present procedure.

Pedicle screw and rod fixation system has been continually modified. Polyaxial screw system was designed for a more secure holding by permitting better contact between the screw head and the rod. Compared with monoaxial pedicle screw, polyaxial screw constructs have some potential biomechanical advantages. Polyaxial screw construct can reduce the incidence of screw breakage [30]. In contrast to a failure pattern extending into the pedicle screw shaft for monoaxial screw, the site of polyaxial screw failure is located at the head coupling to the screw shaft, which can decrease theoretical neurologic risk [31]. Polyaxial screw did not significantly decrease the stiffness of the screw and rod constructs but could increase the resistance to torque by improved rod purchase [32]. In our series, slip correction and the mean slip angle was 71.80 ± 19.38% and 6.39 ± 3.16° after surgery, respectively. During more than 5 years follow-up, they changed to 71.35 ± 12.64% and to 7.21 ± 3.05°, respectively. Loss of reduction was not significant using polyaxial screw construct.

Reduction of a spondylolisthesis is appealing. Reduction could lead to recovery of segmental imbalance and restoration of the original neuroforaminal morphology. Regarding surgical treatment of degenerative spondylolisthesis, the effectiveness of slipped vertebral reduction remains controversial. Several studies analyzing the impact of the reduction of slippage on the clinical outcome showed no correlation between radiological and clinical values assessed with VAS and ODI score [33]. In aged patients with degenerative spondylolisthesis, better radiological outcomes by intentional reduction may not necessarily indicate better clinical outcomes. [34]. However, a quality of life scores study indicated the need to restore physiological alignment of instable and slipped vertebrae in degenerative spondylolisthesis as much as possible. Repositioning of anterior slippage is associated with significantly better results on quality of life assessment [35]. Therefore, whether there is a true beneficial effect of reduction on clinical outcome should be studied further.

During surgical treatment of lumbar spondylolisthesis, reduction is always associated with benefits and risks. For adult low-grade degenerative lumbar spondylolisthesis, the slip is usually mild and is not accompanied with dysplastic changes and lumbosacral kyphosis and reduction is feasible, safe, and easy to achieve. In our reduction procedure, broad and complete decompression of neural elements is recommended in order to avoid neurological complications. Reduction of slippage could effectively relieve clinical complaints and reconstitute physiological spinal load bearing and spino-pelvic balance. Stabilization could eliminate segmental instability and improve fusion conditions. In the current series, satisfactory clinical outcomes were shown according to the data of VPAS pain scores and Oswestry Disability Index for reduction using polyaxial screw and rod fixation system.

## Conclusion

Lever slip reduction maneuver using polyaxial screw and rod fixation system through a single posterior surgical approach was simple, safe, and reliable method for the management of lumbar spondylolisthesis with spinal stenosis. This surgical procedure had low intraoperative blood loss and short operation time. Satisfactory radiographic characteristics and clinical results were shown during mid- and long-term observation.
